# Human NK cells, their receptors and function
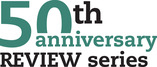


**DOI:** 10.1002/eji.202049028

**Published:** 2021-05-10

**Authors:** Linda Quatrini, Mariella Della Chiesa, Simona Sivori, Maria Cristina Mingari, Daniela Pende, Lorenzo Moretta

**Affiliations:** ^1^ Department of Immunology IRCCS Bambino Gesù Children's Hospital Rome Italy; ^2^ Department of Experimental Medicine University of Genoa Genoa Italy; ^3^ Immunology Laboratory IRCCS Ospedale Policlinico San Martino Genoa Italy

**Keywords:** Adaptive NK cells, Human NK cells, Inhibitory checkpoints, NK cell receptors, TLR

## Abstract

NK cells are cytotoxic components of innate lymphoid cells (ILC) that provide a first line of defense against viral infections and contribute to control tumor growth and metastasis. Their function is finely regulated by an array of HLA‐specific and non‐HLA‐specific inhibitory and activating receptors which allow to discriminate between healthy and altered cells. Human NK cells gained a major attention in recent years because of the important progresses in understanding their biology and of some promising data in tumor therapy. In this review, we will outline well‐established issues of human NK cells and discuss some of the open questions, debates, and recent advances regarding their origin, differentiation, and tissue distribution. Newly defined NK cell specializations, including the impact of inhibitory checkpoints on their function, their crosstalk with other cell types, and the remarkable adaptive features acquired in response to certain virus infections will also be discussed.

## Graphical abstract

During development from hematopoietic stem cells, human NK cells acquire a repertoire of activating and inhibitory receptors that regulate NK‐cell function in different tissues, contributing to immunity. Environmental stimuli, such as viral infections, can impact NK‐cell differentiation favoring the emergence of specialized NK cells, whereas tumor‐derived factors dampen their responses.

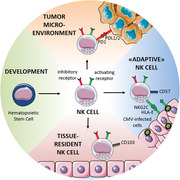

## Introduction

The high pressure exerted by pathogens has been the main driving force in the evolution of the immune system. Thus, from mechanisms based primarily on phagocytosis, the primordial defensive cells acquired receptors capable of selectively sensing pathogen‐associated structures, allowing discrimination between self and infectious non‐self [1]. The acquired ability to produce soluble factors, such as cytokines and chemokines, further improved the defensive capability of such specialized cells allowing the recruitment of and the interaction with other cell types and the induction of inflammatory responses [2]. A further fundamental evolution of the immune system was the acquisition of rearranging genes allowing the production of clonally distributed receptors by subsets of lymphoid cells, ensuring a more fine and efficient recognition of microbial structures [3]. Notably, the primordial defensive cells did not disappear, but rather coevolved with the adaptive immunity playing a synergistic defensive role in contemporary vertebrates. For example, DC became the most efficient APC for T‐lymphocyte activation and the Fc‐gamma receptors (FcγR) evolved from primitive proteins into receptors specific for IgG antibodies allowing a more efficient lysis or phagocytosis of many different pathogens [4]. Regarding NK cells, in addition to FcγRIIIA (CD16), they acquired other molecules, namely killer Ig‐like receptors (KIR), that coevolved with HLA class I molecules and play a fundamental role in NK cell function and in their ability to detect and kill tumor or virally infected cells [5]. While NK cells have been known since mid '70s, other innate lymphoid cells (ILC) sharing with NK cells a common lymphoid precursor, were discovered only recently [6]. Different from NK cells, the other ILCs are mostly confined to mucosal tissues and are noncytolytic. On the basis of transcription factors expressed and of the set of cytokines secreted, these “helper” ILC have been grouped into ILC1, ILC2, ILC3, and LTi. In general, they play both a defensive role at the mucosal barriers and are also involved in tissue repair and homeostasis [6].

As for other cells of the innate immunity, NK cells may acquire a memory‐like function that allows more efficient responses to a second microbial challenge. As discussed below, NK cells display a particularly sophisticated response to CMV, reminiscent of a true T cell‐mediated memory [7].

In this review, we delineate the main characteristics of human NK cells, their origin and development, their receptors, markers, tissue distribution, and functional properties. The subversion of NK cell activities induced by tumors and tumor microenvironment (TME) will be also discussed.

## NK cell development

Human NK cells originate from multipotent HSCs in the BM. According to a classical “linear” model [8], NK cell development occurs through discrete steps, each characterized by the expression of specific surface markers that define a progressive commitment to the lymphoid/NK cell lineage. CD34^+^CD45RA^+^ HSCs in the BM represent the “stage 1,” while the following stages are found in the secondary lymphoid tissues (SLTs: tonsils, lymph nodes, spleen) [9], the principal sites of NK cell development. These stages are defined by the acquisition of CD56, CD117, and IL‐1R1 (stage 2), the loss of CD34 (stage 3) [10], and the downregulation of CD117 with the acquisition of CD94, which marks commitment to the CD56^bright^ NK cells (stage 4) [8]. From this stage on, NK cells express high levels of the transcription factors T‐BET and EOMES. Notably, stage 4 has been further subdivided into stages 4a and 4b, the latter characterized by NKp80 expression and acquisition of cytotoxic capabilities through the formation of cytoplasmic lytic granules containing perforin and granzymes [11]. The exocytosis of these granules occurs at the immunological synapse, allowing NK cells to precisely target the cytolytic process to a single diseased cell without affecting its neighboring cells. In the subsequent stages of development, NK cells express CD16 and KIRs (CD56^dim^ NK, stage 5), and finally CD57 (stage 6) that marks terminally differentiated NK cells [12, 13]. The CD56^bright^ CD16^–^ NK cells are less abundant in the peripheral blood (PB) compared to the mature CD56^dim^CD16^+^ subset, are poorly cytotoxic and, upon exposure to environmental cues, produce high amounts of the NK cell‐hallmark cytokines Interferon‐γ (IFN‐γ) and TNF‐α [14].

The evidence of NK cell diversity in vivo [15] has put in question this “linear” model of development, suggesting that, because of the plasticity of common precursors, environmental cues can eventually modify NK cell development or favor alternative pathways [16]. In this context, it is now recognized that NK cells not only differentiate from lymphoid, but also from myeloid precursors when cultured with NK‐supporting cytokines. This possibility was shown in vitro in cultures from CD34^+^ HSC isolated from cord blood [17, 18, 19] and in vivo in humanized mice [20]. Moreover, a Lin^–^CD34^+^DNAM1^bright^CXCR4^+^ progenitor with NK and T‐cell restricted potential has been identified in the BM [21]. Its marked increase in the PB during chronic inflammatory disorders suggests that it may represent a “shortcut” to rapidly generate effector lymphocytes in antiviral response [21]. Indeed, NK cells arising from this precursor were capable of controlling CMV infection and displayed a mature KIR^+^NKG2C^+^CD57^+^ phenotype [22] that recalls the traits of CMV‐driven NK cell expansions, characterized by memory‐like properties, often referred to as “adaptive NK cells” [23] (see section “NK cell specialization: the “adaptive “ NK cells”).

Importantly, the established NK cell developmental model has been revisited in light of the discovery of noncytotoxic helper ILCs, to which NK cells are related. A progenitor cell population analogous to the “stage 3” NK cell precursor and dependent on the expression of the transcription factor RORγt was identified in SLTs [24, 25], and it was shown that it is indeed capable of generating all human ILC subsets [10]. In the PB, an ILC precursor (ILCP) displaying properties in common with the precursor previously found in SLTs has been identified [26]. This circulating Lin^–^CD117^+^ cell population originates from CD34^+^ HSCs and is enriched in multipotent ILCPs that can give rise to all ILC subsets including NK cells [26]. It was shown that the expression of CD56 by this progenitor marks the divergence of a shared NK/ILC3 common developmental pathway from ILC2s [27]. In addition, in light of the developmental relation between NK cells and helper ILCs, novel functions have been identified for receptors expressed during ILC development. It was shown that NKp46 is a marker that defines the ILC3‐potential, while KLRG1 expression indicates a bias towards ILC2 [28]. Moreover, an additional role has been identified for 2B4 expression on ILC progenitors in vitro: while it was previously shown that it could deliver inhibitory signals during NK cell maturation to spare CD48^+^ bystander precursors [29], it has recently been demonstrated that 2B4 triggering on ILC progenitors favors ILC2 development [30].

**Figure 1:**
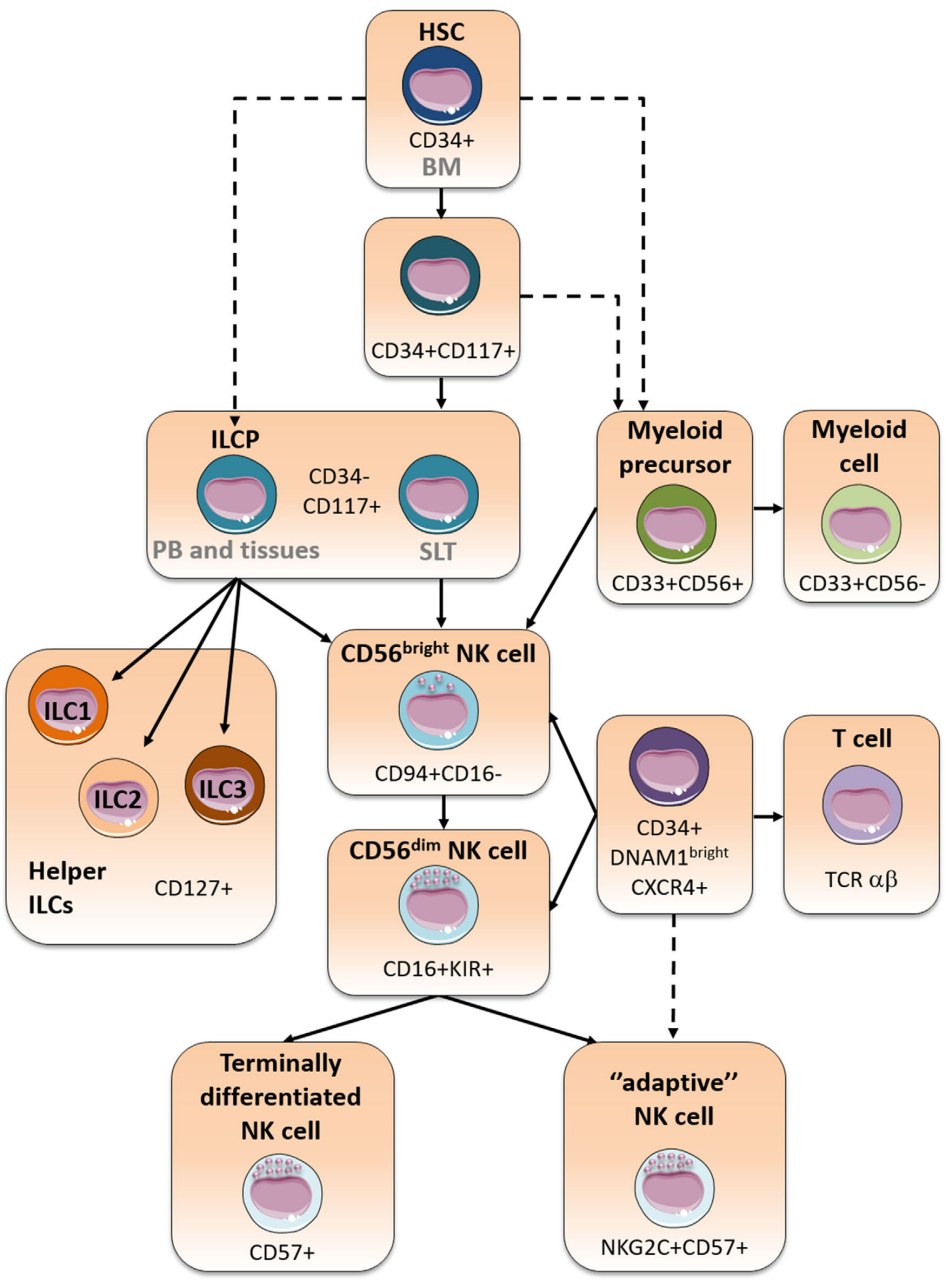
Model of “branched” NK cell development. Starting from HSC in the BM (CD34^+^ cells), NK cell development proceeds by steps, each characterized by a peculiar surface phenotype reported for each stage. The linear pathway has been characterized in SLTs and goes through stage 2 (CD34^+^CD117^+^ cells), stage 3 (CD34^–^CD117^+^ cells), stage 4 (CD94^+^CD16^−^CD56^bright^ NK cells), stage 5 (CD16^+^KIR^+^CD56^dim^ NK cells), and stage 6 (the CD56^dim^CD57^+^ terminally differentiated NK cells). In addition, HSC can generate CD117^+^ ILCP that are localized in PB and peripheral tissues and are analogous to stage 3 NK cells in SLT. ILCPs generate NK cells and helper ILCs. Moreover, in vitro and in humanized mice a myeloid precursor has been identified (CD33^+^CD56^+^), which also generates NK cells. Finally, it was reported that a lymphoid precursor characterized by a CD34^+^DNAM1^bright^CXCR4^+^ phenotype residing in the BM is mobilized in the PB in chronic inflammation and is able to generate NK cells and α/β‐T cells, as well as mature NKG2C^+^CD57^+^ “adaptive” NK cells.

It is now clear that NK cell development is a “branched” rather than a “linear” process (Fig. 1). Alongside the classical NK developmental pathway starting in the BM and proceeding in SLTs in steady state, other pathways exist including the so‐called “ILC‐poiesis” from systemic multipotent ILCPs [31]. It remains to be determined which environmental signals are able to shape these differentiation trajectories, and how the proper response and the rapid and localized generation of NK cells in the tissues and in circulation are generated.

## NK cell receptors

**Figure 2:**
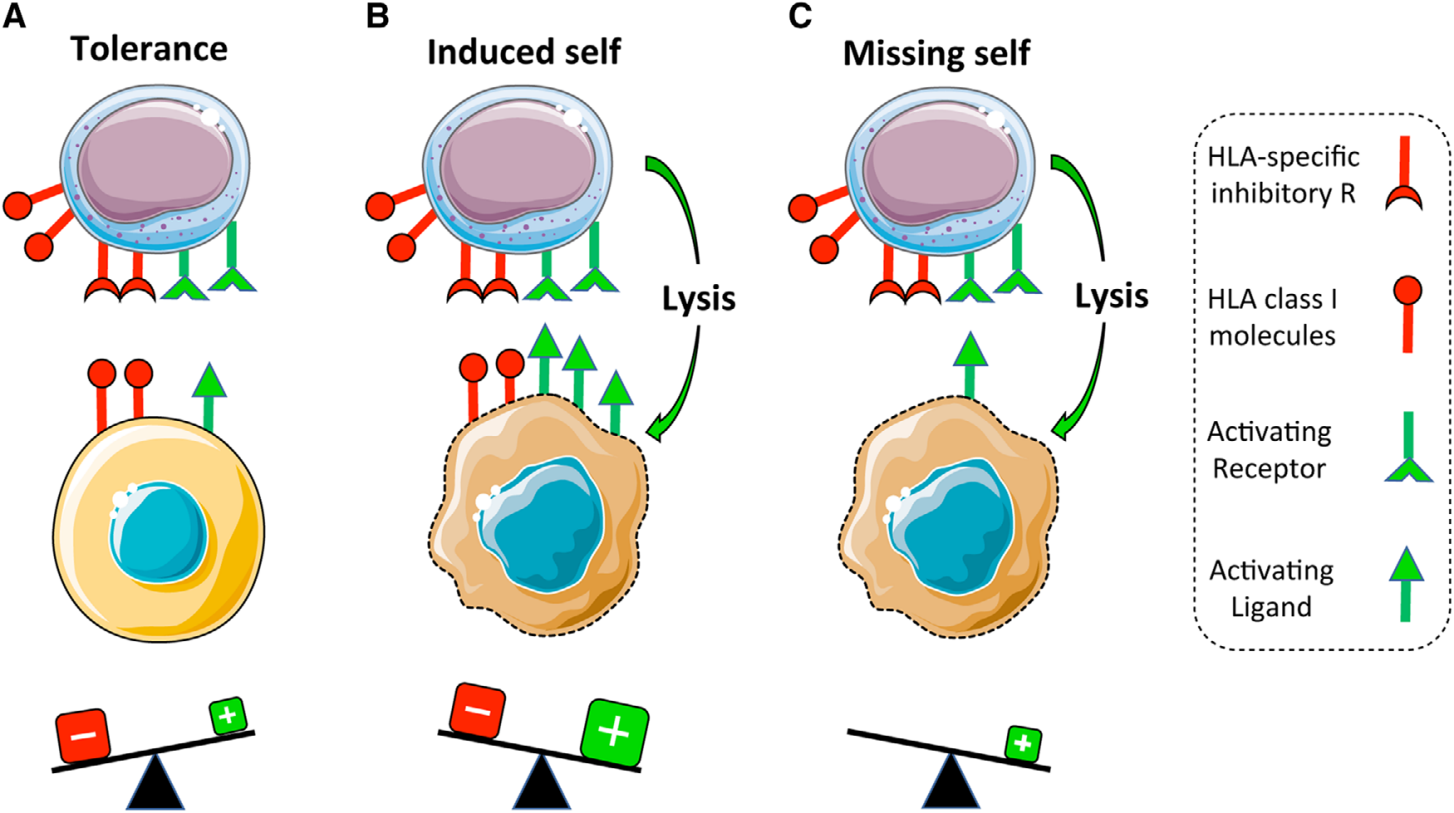
Fine regulation of NK cell function by inhibitory and activating interactions. (A) NK cells are tolerant towards healthy cells, expressing normal levels of ligands (i.e. high HLA class I molecules and low activating ligands), and, thus, inhibitory signals through HLA‐specific inhibitory receptors (KIRs and NKG2A) block activation. The weight of inhibition exceeds activation. (B and C) NK cells can kill pathological (e.g. tumor transformed or virally infected) cells, characterized by overexpression of stress‐inducible ligands (B, “Induced self‐recognition”), increasing the strength of activating interactions, and/or by downregulation of HLA class I expression (C, “Missing self‐recognition”), reducing/abrogating the inhibition mediated by HLA‐specific inhibitory receptors. In both instances, the “on” signal prevails.

NK cells keep in check the health of neighboring cells through several germline‐encoded receptors, either type I proteins of Ig‐like family or type II proteins of C‐type lectin‐like receptor (CTLR) family, upon engagement with specific ligands. The balance between activating and inhibitory signals transmitted by these receptors finely regulates NK cell function (Fig. 2). Crucial for relaying the activating signal is the phosphorylation of ITAMs, more frequently contained within their associated adaptor proteins. The presence of a positively charged amino acid in the transmembrane domain of the receptor allows this association. Conversely, receptors exhibiting inhibitory capacity display a long cytoplasmic tail containing ITIMs, whose tyrosine phosphorylation allows the recruitment of tyrosine phosphatases and transduction of inhibitory signals. In general, inhibitory receptors do not function on their own but regulate the strength of the signal delivered by activating receptors [32].

### Activating NK receptors

“On” signals are crucial for NK cell‐mediated killing of pathological cells overexpressing stress inducible ligands, but also for inducing their physiologic crosstalk with other cell types (Fig. 2A and B). Besides CD16 (FcγRIIIA), that mediates antibody‐dependent cell‐mediated cytotoxicity (ADCC), major activating receptors are natural cytotoxicity receptors (NCRs) and NKG2D [33, 34]. NCRs include NKp46, NKp30, and NKp44 that are Ig‐like transmembrane proteins. NKp46 and NKp30, expressed on virtually all resting NK cells, are associated with CD3‐ζ and/or FcεRI‐γ, whereas NKp44, acquired upon NK cell activation, signals through KARAP/DAP12 adaptor molecule [33]. Functional evidences have documented a primary role of NCRs in NK cell cytotoxicity against various tumor cell types [33] as well as in the crosstalk with other cell types, regulating both innate and adaptive immune responses. An important example is represented by the role of NKp30 in NK/DC interaction mediating the “DC editing” that contributes to the selection of the fittest DCs for antigen presentation and DC maturation [35].

While NCR expression was originally considered to be confined to NK cells, now it is known that NCRs can be expressed by ILCs; expression of NKp44 and NKp46 in a subset of ILC3 has been associated with IL‐22 production, while NCR^–^ ILC3 produce IL‐17 [36]. Moreover, particularly NKp30 has been described on human γδ T and CD8^+^ T cells expanded in IL‐15, which acquire “NK‐like” antitumor activity [37, 38]. Finally, different NCR isoforms, some of which capable to deliver inhibitory signaling behaving as immunosuppressive, have been described and associated with cancer prognosis [39].

**Figure 3:**
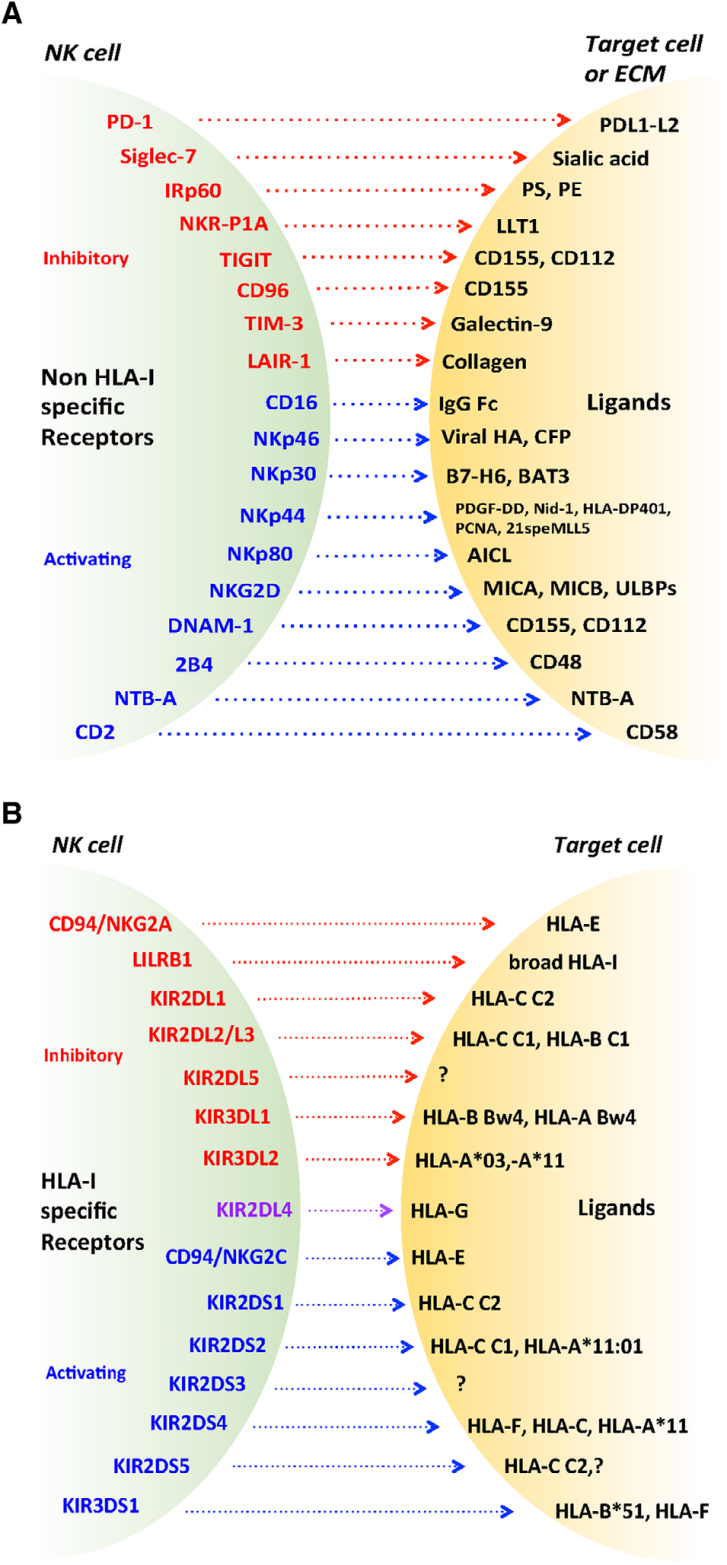
Activating and inhibitory NK receptors and their ligands. (A) NK cells express an array of non‐HLA class I‐ specific activating and inhibitory receptors that recognize ligands usually expressed at the surface of target cells. Certain ligands can also be enzymatically shed and released as soluble ligands, such as B7‐H6, CD155, MICA, MICB, ULBPs, or can be secreted from intracellular compartments, such as BAT3, while further ligands are components of the ECM, such as Nidogen‐1 (Nid‐1) and collagen. PS, phosphatidylserine, PE, phosphatidylethanolamine; HA, hemagglutinin. (B) Different HLA class I‐specific inhibitory and activating receptors are expressed by NK cells that can recognize nonclassical HLA class I molecules (e.g. HLA‐E, HLA‐G, HLA‐F) or epitopes shared by distinct groups of HLA‐A, ‐B, or ‐C allotypes (i.e. C1, C2, and Bw4). The ligands of some HLA class I‐specific receptors are still unknown (e.g. KIR2DS3, KIR2DL5). The receptor KIR2DL4 (in purple) can deliver both inhibitory and activating signals. For details on KIRs and their ligands, see Ref. [53]. Inhibitory receptors are depicted in red and activating receptors are depicted in blue in both panels.

Notably, most NCR ligands activate while others inhibit NK cell function or act as “decoy ligands” when released in soluble form. Indeed, NCR ligands include several different host‐ and pathogen‐encoded molecules, such as membrane‐bound or extracellularly soluble glycoproteins (released by secretion or enzymatically shed or conveyed through extracellular vesicles), or nuclear proteins that can be displayed at the cell surface of target cells (Fig. 3A) [36]. NCRs exert a central role in both the antitumor response (e.g. NKp30/B7‐H6 interaction) and in infectious diseases (e.g. NKp46 recognizes influenza virus hemagglutinins). NCR ligands are also being studied as possible biomarkers in a variety of pathological conditions. Indeed, high levels of soluble NCR ligands have been detected in the sera or in the peritoneal fluid of patients affected by different solid tumors [36].

NKG2D, expressed by NK cells and cytotoxic T cells, is a homodimeric CTLR that transduces the activating signal through DAP10 adaptor protein. NKG2D recognizes MICA/B and ULBPs that are upregulated in virally infected, stressed, and tumor cells [34, 40, 41]. Remarkably, soluble forms of NKG2D‐L shed from tumor cells may be involved in tumor escape mechanisms [42].

NK cells are also equipped with several activating coreceptors including DNAM‐1, NKp80, 2B4, and NTB‐A [43]. The engagement of DNAM‐1 by PVR and Nectin‐2 has been described to be relevant for NK cell killing of various tumors [44], as well as “unfit” DC [45], but also playing a regulatory role in the interaction between decidual NK and trophoblastic cells [46]. NKp80‐AICL interaction promotes an activating crosstalk between monocytes and NK cells in the presence of inflammatory cytokines, leading to the secretion of IFN‐γ and TNF‐α, also playing a role in NK‐mediated surveillance of myeloid leukemia cells [47]. Both 2B4 and NTB‐A belong to signaling lymphocyte activation molecule (SLAM) family receptors (SFRs), and are involved in interactions with hematopoietic cells. Upon receptor engagement, the immunoreceptor tyrosine‐based switch motifs, present in their cytoplasmic tail, become phosphorylated and associate with SLAM‐associated protein (SAP), that initiates the activating signaling pathway. In the absence of SAP, as it occurs physiologically in immature and in decidual NK cells and pathologically in NK and T cells of XLP1 patients (carrying mutations in *SH2D1A* encoding SAP), SFRs associate with tyrosine phosphatases, thus, delivering inhibitory signals [48]. This inhibitory function in XLP1 is considered crucial for the inability to kill EBV‐infected B cells with dramatic clinical sequelae [48].

### HLA‐specific receptors, NK cell education and repertoire

The first HLA‐specific NK cell receptor (NKR) expressed during differentiation is represented by the CTLR heterodimeric CD94/NKG2A. This receptor recognizes the nonclassical HLA‐E, characterized by limited polymorphism, whose surface expression depends on binding nonameric peptides cleaved from the leader sequences of HLA‐A, ‐B, or ‐C [49]. Thus, the interaction between NKG2A and HLA‐E leads to NK cell inhibition toward all HLA class I^+^ cells. The activating counterpart is CD94/NKG2C, whose expression characterizes “adaptive” NK cells (see section “ NK cell specialization: the “adaptive “NK cells”). Like other lymphoid and myelomonocytic cells, NK cells express LILRB1 (also named LIR1 or ILT2) inhibitory receptor, characterized by a broad specificity for HLA [50]. Major NKRs expressed by mature NK cells are KIRs, characterized by 2 or 3 Ig‐like extracellular domains, which include both inhibitory (iKIR) and activating receptors (aKIR). The iKIR (i.e. KIR2DL, KIR3DL), have a long cytoplasmic tail containing ITIMs, while the aKIR (i.e. KIR2DS, KIR3DS) have a short cytoplasmic tail and transduce the activating signal through KARAP/DAP12 adaptor molecule. The iKIR recognize epitopes shared by distinct groups of HLA‐A, ‐B, or ‐C allotypes (termed KIR‐ligands, KIR‐L) (Fig. 3B) [5, 51, 52]. The actual view of KIR/KIR‐L interactions appears more and more complex, underlining the relevance of *KIR* allelic polymorphism and the diverse repertoire of peptides bound to the polymorphic HLA class I molecules [53]. Peptide dependency appears particularly crucial for aKIR/KIR‐L, highlighting a role of KIR2DS1, KIR2DS2, and KIR3DS1 in the response against viruses [54, 55, 56, 57], and KIR2DS4 against bacteria [58].

*KIR* gene family maps in the leukocyte receptor complex on chromosome 19p13.4. Two broad haplotype groups exist: group A with fixed and limited number of genes mainly encoding iKIR, and group B with a greater gene content diversity and rich of genes encoding aKIR [59]. The variability in gene content and allelic polymorphism contribute to the great diversity in *KIR* genotype between individuals [59]. During NK cell development, NK cells undergo a process termed “education,” ensuring both self‐tolerance and the capacity to kill pathological cells that have lost HLA class I molecules (“missing‐self recognition”) (Fig. 2C) [60]. Although different models had been proposed, called “licensing” [61, 62] or “disarming,” [63] they have been reconciled in the concept that NK cells become functionally competent only if they express at least one iNKR specific for self‐MHC class I [64]. NK cell effector function is calibrated by the strength of these inhibitory interactions through a tunable rheostat [65]. This applies not only to the polymorphic iKIR/KIR‐L interactions but also to the conserved CD94/NKG2A recognition of HLA‐E. Indeed, it has been demonstrated that a dimorphism in *HLA‐B* leader sequence at residue −21 encoding either a strong binding methionine (−21 M) and a weak binding threonine (−21 T) determines the variability in HLA‐E expression, and consequently NKG2A^+^ cells from individuals carrying at least one −21 M *HLA‐B* alleles are more efficiently educated [66]. Interestingly, in XLP1 patients, NK cell repertoires, including self‐HLA‐specific iNKR^neg^ NK cells with full effector function have been documented, suggesting the involvement of inhibitory 2B4 and NTB‐A in NK cell education. These cells can exert autoreactivity against mature DC (CD48^neg^), likely exacerbating the patient's immune dysfunction [67].

A highly variegated receptor repertoire of the circulating NK cell pool can be detected among different donors, primarily due to the high polymorphism of the coinherited, but independently segregating, *KIR* and *HLA* class I genotypes [59]. Notably, KIR expression pattern is the result of a stochastic event but is highly influenced by self‐HLA class I molecules. High efficiency NK cell cloning demonstrated the clonal expression of each NKR, and the various patterns of coexpression at the single‐cell level [5]. This approach was fundamental to demonstrate that certain NK cells can be alloreactive, defined by the unique expression of iKIR specific for self‐HLA class I molecules (KIR‐L), missing instead in allogeneic HLA repertoire [68]. Nowadays, advanced multiparametric flow cytometry and mass cytometry by time‐of‐flight technologies allow the concomitant assessment of many parameters, revealing more than 10^5^ phenotypically distinct subsets in each individual [15]. In addition to genetics, environmental factors, in particular viral infections, can impact on the NK cell repertoire (see section “NK cell specialization: the adaptive “NK cells”).

Remarkably, NKG2A and KIR may be expressed also by T lymphocytes and compromise their function in vitro [69], as recently shown for NKG2A also in tumor‐infiltrating T cells in both humans and mice [70]. Combination therapies, including antibodies blocking NKG2A or KIRs immune checkpoints (ICs), appear efficacious in clinical trials [71].

### Non‐HLA‐specific inhibitory receptors

NK cells also express additional inhibitory receptors that recognize cell surface or extracellular ligands (Fig. 3A), regulating the strength of activating receptors and contributing to the physiological control of immune responses and tolerance. In pathological conditions, as during viral infections and in the TME, some of these receptors can be upregulated and/or de novo expressed and inhibit responses against infected or tumor cells. These inhibitory interactions can limit exacerbated immune responses but also facilitate tumor escape. In this context, they represent true IC which, in immunotherapy, can be targeted by IC inhibitors (ICI). The prototypic IC is PD‐1 that binds ligands (PD‐L) expressed on many solid and hematologic tumor cells, on infected cells, on APCs in inflammatory foci, and in SLTs. While PD‐1 is virtually absent on PB NK cells from healthy individuals, PD‐1^+^ NK cells have been detected at tumor sites [72, 73, 74]. Thus, PD‐1/PD‐L1 inhibitory interactions represent a major immune escape mechanism in the TME. Additional IC are TIGIT and CD96, reacting with PVR (CD155) and Nectin‐2 (CD112), thus, competing with DNAM‐1 [75]. Moreover, TIM‐3 is considered a marker of NK‐cell exhaustion in advanced tumors, and its inhibitory activity can be reversed by blocking antibodies in humans [76]. Other relevant inhibitory receptors include NKR‐P1A/CD161, Siglec‐7/CD328, LAIR‐1/CD305, and IRp60/CD300a. NKR‐P1A, expressed early during NK cell maturation, inhibits NK cell cytotoxicity and cytokine secretion upon binding with LLT1 glycoproteins expressed on activated B cells [77] or on various tumors [78]. Siglec‐7, mostly confined to NK cells, upon recognition of sialic acids can contribute to immune tolerance and also decrease the antitumor activity, also considering the hypersialylation as a tumor escape mechanism. Notably, Siglec‐7 downregulation can be an early marker of dysfunctional NK cells, associated with CD56^neg^ phenotype and an aberrant activating and inhibitory NK receptor repertoire in patients with high levels of HIV‐1 viremia [79]. Low/negative Siglec‐7 and NKR‐P1A expression characterize also certain CMV‐driven adaptive hallmarks [13, 80]. Finally, LAIR‐1, binding collagens, and IRp60, recognizing the phosphatidylserine and phosphatidylethanolamine aminophospholipids, are broadly expressed on hematopoietic (both lymphoid and myeloid) cells, thus, displaying a wide ability to modulate immune functions and participation in the host response to different pathologies including infectious diseases, cancer, allergy, and chronic inflammatory diseases [81, 82].

### The pathogen‐recognizing receptors TLRs the negative regulator IL‐1R8

TLRs are germline encoded pattern‐recognition receptors that are essential for the recognition of invading pathogens, triggering of innate responses, and shaping of subsequent adaptive immune responses [83]. Different functional TLRs are expressed on various cells of the innate immunity including NK cells. In particular, several studies reported that TLRs (i.e. TLR2, TLR3, TLR5, TLR7/8, and TLR9) are capable of inducing NK cell activation leading to increased cytotoxicity and cytokine production [84]. However, efficient NK activation by PAMPs occurs preferentially thanks to complex interactions with other innate cells providing the chemokines/cytokines microenvironment required for optimal NK cell response. Furthermore, TLRs signaling contributes to the NK cell‐mediated editing of maturing DC, a “quality control” process by which NK cells select mature DCs able to mediate optimal T‐cell priming in SLTs [85]. Moreover, a peculiar TLR/KIR cooperation was revealed in human NK cells. Indeed, KIR3DL2 can bind CpG‐ODNs (TLR9 ligands) at the NK cell surface and shuttle them to endosomes where interaction with TLR9 triggers NK cell activation [86].

Interestingly, a two‐way regulation between TLRs and miRNAs has recently been described primarily in macrophages. Indeed, TLR signaling modulates miRNA expression, whereas some miRNA regulate TLR expression/signaling or induce immune and inflammatory gene expression upon binding to TLR3 and TLR7/8 [87]. It will be interesting to evaluate whether these events occur also in NK cells.

In addition to TLRs, members of interleukin‐1 receptors (ILRs) family are key players in innate immune responses and inflammation. Similar to TLRs, they are characterized by the presence of the Toll/Interleukin‐1 receptor (TIR) domains in their cytoplasmic tail. Binding of agonists to the ectodomain of the TLRs or ILRs induces the association of their TIR domains with adaptor polypeptides involved in activation of protein kinases and, consequently, of transcription factors and genes involved in immune responses [88]. Recently, IL‐1R8, a member of the ILR family, was proposed as negative regulator of NK cells. Indeed, its deficiency associates with higher numbers of mature NK cells in different tissues, higher expression of activating NK receptors, increased production of IFN‐γ, granzyme B content, and degranulation. These effects were abrogated after IL‐18 depletion or in IL‐1R8/IL‐18 double‐deficient mice, thus, demonstrating that IL‐1R8 modulates the signaling of IL‐18, a cytokine relevant for NK cell activation [89]. In addition, IL‐1R8 inhibits activation of NFkB and JNK induced by TIR‐mediated signaling upon ILRs or TLRs engagement with their ligands [90]. Interestingly, the negative regulation of TLR signaling represents a strategy of immune evasion adopted by several pathogens [91, 92]. Indeed, in some bacterial infections, TIR‐containing proteins interfere with TIR‐TIR association, sharing with IL‐1R8, the ability to interfere with TIR‐containing signaling receptors. IL‐1R8 blockade in NK cells has been proposed as a novel therapeutic approach to unleash NK cells against solid tumors [89]. In this regard, also some TLR agonists are under investigation for treatment of solid tumors [93].

## NK cell specialization: the “adaptive” NK cells

Through a branched developmental pathway, NK cells arise and progressively evolve from a limited diversity status at birth to highly differentiated and variegated phenotypes, characterized by various receptors repertoire and function, thus, achieving “a broad spectrum of diversity” [94], which is dictated both by genetic factors and largely by environmental stimuli such as pathogen exposure [15, 94, 95].

One of the most striking examples of influence exerted by pathogen challenge is the profound effect induced by CMV on NK cells [7]. This herpes virus has revealed as a key driver of NK cell differentiation and specialization by inducing a long‐lasting imprinting in NK cell receptor repertoire as well as enhanced effector function, suggesting that innate lymphocytes can share some memory properties with adaptive lymphocytes [96]. Indeed, all innate immune cells are able not only to rapidly sense and respond to pathogens or their products, but also to increase their responsiveness to a second exposure to the same pathogen. This phenomenon represents the so called “trained immunity” or innate immune memory, which is mostly based on epigenetic modifications and has been mainly studied in monocyte/macrophages. Notably, trained immunity is usually not Ag‐specific and shows limited persistence [97, 98, 99]. However, the profound modulation induced by CMV on NK cells is far beyond a trained immune response. Indeed, upon CMV infection, NK cells undergo a major reconfiguration resulting in the expansion of NK cells expressing the HLA‐E‐specific activating receptor CD94/NKG2C and displaying a highly differentiated surface signature, namely, self‐KIR^+^NKG2A^–^LILRB1^+^CD57^+^Siglec7^−^ [100]. This CMV‐driven NK cell population, termed “adaptive,” is characterized by stable epigenetic modifications that regulate both surface receptors and expression of signaling adaptor proteins, thus, tuning NK cell responsiveness [13, 101]. Adaptive NK cells are poor responders to IL‐12 and IL‐18 and express the NCRs NKp30 and NKp46 at low levels at variance with conventional mature NK cells (non‐ CMV‐imprinted). On the other hand, they respond very efficiently to NKG2C triggering by releasing cytokines (e.g. IFN‐γ and TNF‐α), cytotoxic molecules and undergoing proliferation despite their highly differentiated status [13, 80, 101, 102]. In addition, adaptive NK cells are endowed with increased ADCC ability, which is, at least in part, due to the (epigenetically controlled) downregulated expression of the signaling molecule FcεRI‐γ, occurring in a consistent fraction of adaptive NK cells detected in CMV^+^ healthy subjects [13, 80]. By supporting Ab‐mediated responses through CD16 engagement, adaptive NK cells can keep under control also infections caused by viruses other than CMV, as suggested by studies reporting their efficient ADCC‐mediated killing of opsonized EBV‐ and HSV‐infected cells [103, 104]. In this context, adaptive NK cells could contribute also to the protective effect of certain vaccinations, although their highly differentiated signature could limit their responsiveness to vaccine products [105]. Of note, Maucourant et al. have recently described different PB NK cell immunotypes in COVID‐19 patients, linking the presence of strongly activated adaptive NK cells to severe disease [106]. Moreover, adaptive NK cells have been shown to express checkpoint receptors, such as LAG‐3 and PD‐1, upon chronic stimulation through activating receptors engagement [107]. This phenomenon, reminding of T cell‐responses, could render adaptive NK cells hypofunctional in certain circumstances.

In line with their specialized condition, recent reports have demonstrated that CMV‐induced NK cells can distinguish subtle differences among peptides bound to HLA‐E molecules reminiscent of Ag‐specific T‐cell responses [108, 109]. Furthermore, in transplantation, adaptive NKG2C^+^CD57^+^ developing upon CMV reactivation revealed an unusual longevity, persisting up to 2 years [102, 110, 111], thus, definitively challenging the conventional view of NK cells as short‐lived innate immune cells, unable to keep memory. However, open questions remain regarding the actual role of NKG2C in driving the generation of adaptive NK cells, in view of the consistent expansions of adaptive NK cells also in individuals lacking the NKG2C gene [112, 113]. Indeed, an alternative role in driving anti‐CMV responses has been proposed for aKIRs and/or CD16 in cooperation with the coreceptor CD2 [114]. The involvement of aKIRs is in line with previous observations in mice where the expansion of a NK cell subset expressing the activating receptor Ly49H, homologue of aKIR, in response to MCMV infection, was originally described [115]. However, while in mice Ly49H^+^ NK cells recognize the viral‐encoded ligand m157 and confer long‐term protection to secondary challenges, in humans the exact mechanisms underlying the recognition of infected cells by aKIRs are not entirely clear, although a role for KIR2DS1 in the recognition of its ligand HLA‐C2, modified by CMV in infected fibroblasts, has been reported [54].

It is also still unclear which are the exact signals provided by CMV infection, capable of remodeling NK cell epigenetic landscape. Together with the engagement of activating receptors and coreceptors, the exposure to proinflammatory cytokines (e.g. IL‐12, IL‐18, IL‐15), available in an inflammatory milieu, seems crucial to drive the expansion of specialized subsets [109]. Indeed, exposure to proinflammatory cytokines can induce epigenetic remodeling of the IFNG conserved noncoding sequence 1, favoring IFN‐γ production by NK cells upon receptor engagement or cytokine stimulation [116]. Along this line, preactivation of NK cells with IL‐12, IL‐15, and IL‐18 confers enhanced effector function after restimulation, inducing a cytokine‐mediated memory‐like (CIML) response [117]. Cytokine‐primed CIML‐NK cells represent a promising tool in cancer immunotherapy [118], however, their imprinting is quite different from that shown by CMV‐driven memory NK cells, being neither Ag‐specific nor phenotypically skewed [117]. It cannot be excluded that the specialized status achieved by CMV‐induced NK cells requires not only the simultaneous triggering of given activating receptors by selected peptides in the presence of proinflammatory cytokines but also the recognition of CMV‐derived PAMPs through TLRs. In this context, NK cells can directly recognize CMV virions through TLR2, which can bind gB and gH glycoproteins from the CMV envelope [119], and through TLR9 that can bind viral DNA, receiving activation and priming signals [85].

Intriguingly, the generation of such specific NK cell subsets could rely also on the release of particular committed precursors that exit the BM in inflamed conditions and undergo rapid differentiation toward adaptive NKG2C^+^KIR^+^ NK cells responding to CMV, as recently reported in humans [120]. Whether these alternative differentiation trajectories could favor the prompt emergence of specialized NK cell subsets specific for other pathogens is unknown and deserves further investigation.

In conclusion, NK cells are able to adapt to their surroundings undergoing phenotypic and functional changes in response to cytokines, chemokines, and other soluble factors, such as PAMPs and/or to receptor engagement, showing a high degree of both functional and developmental plasticity, as explicitly demonstrated by the emergence of virus‐driven adaptive NK cell subsets.

## Tissue‐resident NK cells

NK cells continuously circulate among blood, LNs, and tissues to rapidly recognize and eliminate “altered” cells such as those infected by viruses. In the PB, NK cells comprise 5 to 20% of lymphocytes and are the most studied NK cell subset. However, NK cells can also be resident in tissues and acquire a specific phenotype and function that allows them to patrol the local environment. In multiple organs, including liver, lung, skin, kidneys, and BM, subsets of NK cells expressing markers of tissue residency, such as CD69 and CD103, have been identified and are considered to be true “tissue‐resident” NK cells (tr‐NK cells), distinct from circulating NK cells. The definition of tr‐NK cells has been challenged by the discovery and characterization of ILC1s residing in tissues, which have revealed a considerable heterogeneity and opened the question of whether these tr‐NK cells and ILC1s may correspond to different developmental and/or activation states imprinted by the microenvironment on the same lineage [121].

In a recent study, a high‐dimensional transcriptional and phenotypic profiling of NK cells across multiple tissues within individual donors has been performed [122]. It was shown that NK cells in blood, BM, spleen, and lung are predominantly CD56^dim^CD16^+^ and are present at higher frequencies compared to LNs, tonsils, and gut where NK cells are mostly CD56^bright^. It was also demonstrated that, although NK cell subset (CD56^bright^ vs CD56^dim^) identity is preserved across sites, tissue localization drives further subset‐specific transcriptional programs. Moreover, multiple states of NK development and differentiation have been detected across blood and tissues, with LNs containing the most immature subsets, and blood and lung containing mature and more differentiated states [122]. The tissue‐intrinsic modes of NK cell maintenance, differentiation, and functional regulation revealed in this study suggest a model of anatomical compartmentalization in which lymphoid tissues and intestine represent precursor and immature NK cell reservoirs, while immunosurveillance and effector functions would occur primarily in the blood, spleen, and lung, where activated and differentiated NK cells are located. The liver is another organ constantly challenged by foreign antigens drained from the gut, and NK cells are responsible for immunosurveillance also at this site. tr‐NK cell retention in the liver is mediated by CXCR6, CXCR3, and CCR5, which interact with chemokines released by stromal cells [123, 124]. Liver tr‐NK cells are enriched in CD56^bright^ NK cells and are characterized by a peculiar transcription factor profile that includes HOBIT, T‐BET, high EOMES expression and by a TIGIT^+^CD69^+^CXCR6^+^CD49e^–^ phenotype [125, 126]. Another organ enriched in NK cells is the uterus. NK cell frequency sharply increases in the decidua after embryo implantation reaching 40–70% of total lymphocytes during the first trimester of pregnancy [127]. This tr‐NK cell subset is responsible not only for the defense against infections, but also for the establishment of the maternal‐fetal tolerance [128]. Decidua NK cells are characterized by a CD56^bright^CD16^−^KIR^+^CD9^+^CD49a^+^ phenotype, are poorly cytolytic, produce low amounts of IFN‐γ, and are specialized in the secretion of cytokines and chemokines (VEGF, SDF‐1, IP‐10) that promote neoangiogenesis, tissue remodeling, immune modulation, and placentation [129]. Thus, microenvironment can drive further NK cell specialization in given organs. It is of note that the immunosuppressive milieu of decidua, which here plays a physiologic role, is similar to the microenvironment of different tumors, where it may instead favor tumor growth by suppressing the immune response (see next section).

## NK cell inhibition by the microenvironment

tr‐NK cells display peculiar phenotypic and functional features in specific tissues in physiological conditions. Analogously, in pathological conditions, NK cells may acquire a distinct phenotype and function, in response to signals delivered by both immune and stromal cells present in the microenvironment. The best example of this regulation is given by the immune suppressive effect played by the microenvironment in the tumor context. NK cells isolated from tumors, indeed, display impaired effector functions and a dysfunctional phenotype induced by soluble mediators or by cell‐to‐cell contact within the tumor [130]. This suppressive effect represents a mechanism of “immune evasion” by the tumor, as dysfunctional NK cells are characterized by the downregulation of the main activating receptors involved in tumor recognition (NCRs, DNAM‐1, and NKG2D) and their presence is associated with an adverse clinical outcome [130]. Cancer cells have developed multiple mechanisms to escape cytotoxic immune responses, many of which involve evasion of NKG2D recognition. The presence of NKG2D‐expressing cytotoxic lymphocytes in the TME favors the selection of tumor cell clones releasing soluble NKG2D ligands from their surface through alternative splicing, proteolytic shedding, or exosome secretion [131]. Besides rendering cancer cells “invisible” to NK cell recognition, the release of soluble ligands induces NKG2D downregulation, thus, further impairing NK cell response [132]. In addition to the tumor context, soluble NKG2D ligands were detected in sera of patients with celiac disease and rheumatoid arthritis [133, 134]. However, soluble NKG2D ligands in inflammatory autoimmune conditions triggered direct NK activation with no NKG2D downmodulation, due to high concentrations of proinflammatory cytokines such as IL‐15 and TNF‐α [133, 134]. Therefore, the effect of the microenvironment on NK cell responses is strictly dependent on the context. Analogously, the steroid hormones glucocorticoids (GCs) were identified as indispensable for the transcriptional and translational induction of PD‐1 expression on NK cell membrane [135]. However, although high concentrations of GCs could be detected in both blood and pleural exudate of lung cancer patients, only tumor‐associated NK cells displayed high levels of surface PD‐1 [136]. Indeed, GCs alone are not sufficient for PD‐1 surface expression in NK cells that also requires the combined effect of IL‐15, IL‐12, and IL‐18, present in the TME [135]. Another component of the TME with a potent immune suppressive effect is TGF‐β. Notably, upon exposure to TGF‐β, NK cells might even be converted into ILC1‐like cells that lack the ability to control tumor growth [137]. In addition to TGF‐β, other soluble molecules, such as IL‐10, prostaglandin E2, and metabolites of IDO contribute to suppress NK cell function and render them “tolerant” [130]. Of note, these mediators characterize both the TME [138] and the immune suppressive milieu of decidua (see section “Tissue‐resident NK cells”) [139], but NK cell “tolerance” leads to different outcomes in the two conditions.

Taken together, these data indicate that NK cell response to a given stimulus may depend on the combination of signals concomitantly delivered in given environments. A deeper characterization of these extrinsic mechanisms of regulation of NK cell response may help to design therapeutic strategies complementary to those targeting NK cells directly. In the context of antitumor therapies, for example, blocking the TME components responsible for the downregulation of NK cell response may further contribute to unleashing their antitumor function.

## Concluding remarks and future perspectives

The groundbreaking discovery of HLA class I‐specific inhibitory receptors and the demonstration that they control NK cell activation and function [53] paved the way to the fundamental concept that the immune response is regulated by an array of inhibitory receptors. These receptors represent true checkpoints controlling either individual cells [140] or more complex responses [82]. This knowledge, together with the discovery of receptors mediating NK cell activation, offered clues for major progress in cancer therapy. In this context, the KIR‐dependent donor NK cell alloreactivity has been exploited in the haploidentical HSC transplantation (HSCT) to treat patients with high‐risk leukemias. Of more general interest in tumor therapy has been the use of ICI. Notably, NK cells may play an important role in treatments based on mAbs disrupting the PD‐1/PD‐L axis, particularly in tumors that have lost HLA class I expression, thus, resulting undetectable by T lymphocytes but more susceptible to NK‐mediated attack. Harnessing NK cell function is presently a major issue in novel approaches of tumor immunotherapy. For example, ongoing studies on the diversity of NK precursors, in particular, the identification of novel progenitors capable of rapidly generating mature “adaptive” NK cells displaying strong cytolytic activity and maintaining proliferative capacity, may offer novel important clues. Indeed, favoring the exit of these progenitors from BM and/or their proliferation may provide a tool to generate NK cell progenies efficient not only against CMV, but also “trained” for a pathogen‐agnostic antimicrobial and antitumor resistance. Transfection with IL‐15 gene allows to potentiating NK cell function and prolonged their survival. Other novel approaches are based on the use of engineered soluble molecules (known as BiKE, TriKE, and Engagers), bridging activating receptors, and/or CD16 on NK cells to tumor antigens on cancer cells [71]. A particularly promising approach is the use of NK cells genetically modified with chimeric antigen receptors (CAR‐NK) targeting tumor antigens. Indeed, different from T lymphocytes, NK cells do not cause GvHD. Thus, CAR‐NK cells derived from unrelated donors represent an excellent “off‐the‐shelf” product [71]. The proven capability of growing and storing very high numbers of NK cells allows the prompt treatment of patients, using programmed cell numbers with no need of transfecting T cells from individual patients. Although the TME and/or suppressive cells generated in cancer patients can greatly interfere with most cell‐based immunotherapeutic approaches, the antitumor effect may be rescued by combining therapies (e.g. ICI) targeting major inhibitory mechanisms. In conclusion, NK cells are likely to offer a crucial tool in many of these novel immunotherapeutic approaches.

## Author contribution

All authors have provided intellectual contribution to the work and approved it for publication.

## Conflict of interest

The authors declare no commercial or financial conflict of interest.

**Abbreviations**
ADCC: antibody‐dependent cell‐mediated cytotoxicity
CIML: cytokine‐mediated memory‐like
CTLR: C‐type lectin‐like receptor
GC: glucocorticoid
HSCT: HSC transplantation
IC: immune checkpoint
ICI: immune checkpoint inhibitor
IFN‐γ: Interferon‐γ
ILC: innate lymphoid cell
ILCP: innate lymphoid cell precursor
ILR: interleukin‐1 receptor
KIR: killer Ig‐like receptors KIR‐L KIR‐ligands
NCR: natural cytotoxicity receptor
NKR: NK cell receptor
PB: peripheral blood
SLAM: signaling lymphocyte activation molecule
SFRs: SLAM family receptors
SAP: SLAM‐associated protein
SLT: secondary lymphoid tissue
TIR: TIRtoll/interleukin‐1 receptor
TME: tumor microenvironment
tr‐NK cell: tissue‐resident NK cell
